# 

**Published:** 1891-09-19

**Authors:** 


					INBORNE'S
for INFANTS
ISINGLASS
AND INVALIDS.
No. 260. Vol. X.] Saturday,
September 19th, 1891.
[One Penny.

				

